# Renal Vein Thrombosis Treated With Apixaban in a Patient With COVID-19: A Case Report

**DOI:** 10.7759/cureus.39296

**Published:** 2023-05-21

**Authors:** Tigran Kakhktsyan, Aliaa Mousa, Hassaan Arshad, Kateryna Chepenko, Tehreem Fatima

**Affiliations:** 1 Internal Medicine, Capital Health Regional Medical Center, Trenton, USA

**Keywords:** atypical thrombosis, renal vein thrombosis, doacs, direct oral anticoagulants, covid-19

## Abstract

Renal vein thrombosis (RVT) is a rare condition characterized by the formation of a blood clot in one or both of the renal veins. Bilateral involvement is more common, but when the condition affects only one side, it usually occurs on the left due to more extensive venous vasculature compared to the right side. RVT can be caused by various factors such as trauma, dehydration, malignancy, and a hypercoagulable state. Acute RVT is typically more severe than chronic, and it can cause symptoms such as renal infarction, acute kidney injury, renal failure, severe flank pain, and hematuria. Some cases of RVT have also been linked to coronavirus disease 2019 (COVID-19), which is widely recognized to induce a hypercoagulable state. The standard treatment for RVT is warfarin but in this case report, we describe a COVID-19 patient with RVT whose thrombus was successfully treated with direct-acting oral anticoagulant (DOAC) apixaban for six months.

## Introduction

Coronavirus disease 2019 (COVID-19) is a highly infectious virus that is often associated with respiratory symptoms such as viral pneumonia, but it can also be asymptomatic. However, the virus has been linked to a variety of other pathologies, including coagulopathy. Venous thromboembolism is a common complication in patients with acute COVID-19 infection, specifically those who require hospitalization [1,2]. This can include cases of renal vein thrombosis (RVT), a diagnosis that is typically rare and mostly seen in individuals with nephrotic syndrome. Warfarin is the standard treatment for RVT [3]. In this case report, we discuss the case of a COVID-19 patient who developed RVT and was successfully treated with the direct-acting oral anticoagulant (DOAC) apixaban for a duration of six months to manage the thrombus.

## Case presentation

**Figure 1:**
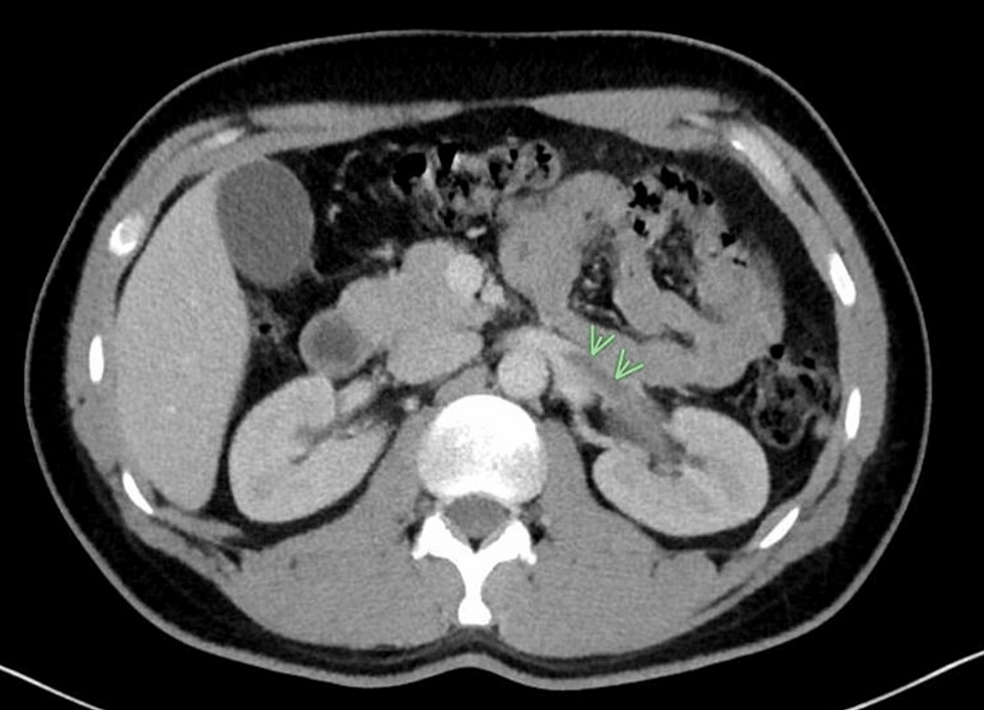
Computed tomography (CT) of abdomen and pelvis with contrast showing thrombosis of the left renal vein (green arrows)

The patient, a 39-year-old male with no significant past medical or family history who had not received the COVID-19 vaccine and had upper respiratory tract infection symptoms including cough, congestion, and fatigue before hospitalization, complained of severe left-sided abdominal pain, vomiting, and dizziness that had been gradually worsening for about a week. Upon arrival at the emergency room, his vital signs were stable, with a blood pressure of 120/76 mmHg, a heart rate was 70 beats/min, a respiratory rate of 18/min, oxygen saturation was 100% on room air, and a body temperature was 37.0°C. Physical examination revealed severe tenderness in the left abdomen and flank. The pain was not relieved by non-steroidal anti-inflammatory drugs (NSAIDs) or Tylenol. A computed tomography scan (CT) of the abdomen and pelvis with contrast was performed and showed acute left RVT (Figure 1).

The coagulation panel results showed no abnormalities, with a normal range partial thromboplastin time (PTT) of 22 seconds, prothrombin time (PT) of 14.5 seconds, and international normalized ratio (INR) of 1.1. The patient's creatinine level was 0.79 mg/dL, blood urea nitrogen was 8 mg/dL, and urinalysis was unremarkable, with no pyuria, proteinuria, or blood found. The patient tested positive for COVID-19. Also, a hypercoagulation panel was performed, which ruled out the most common hypercoagulable syndromes, including factor V Leiden, protein C and S deficiency, antithrombin deficiency, and antiphospholipid syndrome.

**Figure 2:**
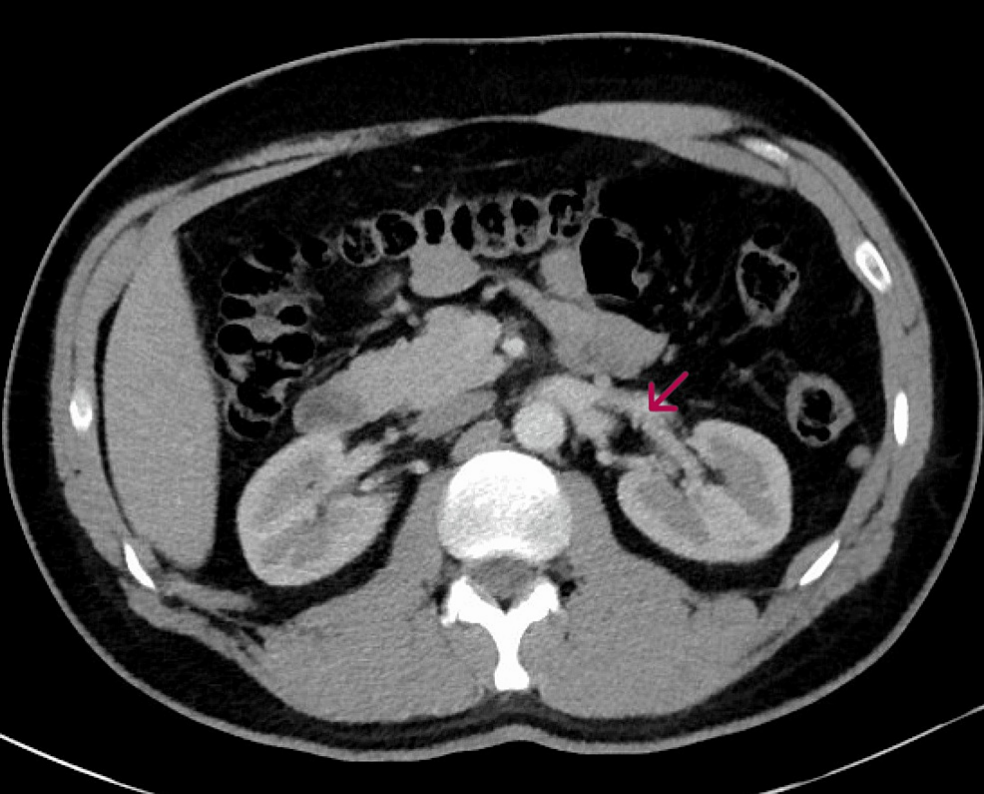
Computed tomography (CT) of abdomen and pelvis with contrast showing resolution of thrombus in the left renal vein after six months of treatment with a DOAC (red arrow) DOAC: direct-acting oral anticoagulant

To treat the RVT, the patient was started on apixaban 5 mg twice daily for six months. After discharge, the patient was followed up at the hospital clinic for six months, during which time he remained symptom-free, and had no signs of kidney injury. A repeat CT of the abdomen and pelvis with contrast was performed six months after apixaban therapy and showed thrombus resolution (Figure 2).

## Discussion

RVT is an uncommon medical condition that is typically caused by nephrotic syndrome but can also be brought on by other factors like inherited hypercoagulability syndromes, such as factor V Leiden mutation and antiphospholipid syndrome, malignancy, infection, or trauma. The condition is more likely to affect the left renal vein than the right, and it's more often seen in men than women. In fact, about two-thirds of patients with RVT will have bilateral renal vein involvement [3]. COVID-19 has also been associated with some cases of RVT, which is attributed to the ability of the virus to cause direct harm to the endothelium and trigger an overactive inflammatory response [1,2]. There have been documented case reports highlighting the occurrence of RVT in patients diagnosed with COVID-19 [4,5].

The most common symptoms of RVT include flank pain, hematuria, fever, nausea, and vomiting, which are nonspecific and often misinterpreted as renal colic [3,6]. Therefore, imaging is necessary for screening and diagnosis. Doppler ultrasound has found broad application as a screening tool in high-risk patients, while CT angiography is the primary modality for diagnosing RVT due to its superior sensitivity and specificity compared to traditional ultrasound [7]. Medical management utilizing anticoagulants has now emerged as the mainstay approach for treating RVT [3].

While warfarin has been the traditional treatment option for RVT, recent studies have suggested that DOACs may also be effective. However, it's worth noting that DOACs were not included in phase III studies for patients with venous thrombosis in non-typical locations such as renal veins [8]. As a result, the current recommendation for such thrombi remains unfractionated or low molecular weight heparin (LMWH) followed by warfarin. Despite this, a few smaller non-randomized studies have reported positive outcomes with DOACs, including apixaban and rivaroxaban, for treating RVT [3,9].

A case of left RVT in a COVID-19 patient was effectively managed using apixaban, resulting in complete thrombus resolution within six months. This outcome was confirmed through CT imaging. This case adds to the growing body of evidence supporting the use of DOACs in RVT treatment and highlights the potential benefits of considering this approach for COVID-19 patients with similar conditions.

## Conclusions

While a few small studies have shown promise, there is still a lack of research regarding the use of DOACs in cases of RVT. Furthermore, the effectiveness of DOACs such as apixaban in treating RVT in patients with COVID-19 has not been studied. Our case illustrates a successful and safe example of using apixaban for this purpose. Nevertheless, it is crucial to conduct further comprehensive research to establish the potential role of DOACs in treating acute RVT, particularly in patients who have COVID-19.
